# Spatial Immune Model of Alveolar Lung Infection (SIMALI) Identifies Structural Determinants of Lung Inflammation

**DOI:** 10.21203/rs.3.rs-9986593/v1

**Published:** 2026-06-24

**Authors:** Humayra Tasnim, Stephanie Forrest, Steven Hofmeyr, Alan M. Friedman, Ronak Etemadpour, Hossein Mehdikhani, Akil Andrews, Judy L. Cannon, Melanie E. Moses

**Affiliations:** 1 Department of Computer Science, University of New Mexico, Albuquerque, 87106, New Mexico, United States.; 2 Department of Molecular Genetics and Microbiology, University of New Mexico, Albuquerque, 87106, New Mexico, United States.; 3 Biodesign Institute, Arizona State University, Tempe, 85281, Arizona, United States.; 4 Santa Fe Institute, Santa Fe, 87506, New Mexico, United States.; 5 Applied Mathematics and Computational Research, Lawrence Berkeley National Laboratory, Berkeley, 94720, California, United States.; 6 Department of Biological Sciences, Purdue University, West Lafayette, 47907, Indiana, United States.; 7 Medical Physics Residency Program, Unio Health Partners, Torrance, 90503, California, United States.; 8 Nuclear Medicine and Molecular Imaging, City Of Hope, Irvine, 92618, California, United States.; 9 Vaccine and Infectious Disease Division, Fred Hutchinson Cancer Center, Seattle, 98109, Washington, United States.

**Keywords:** Agent Based Model, Simulation, Lung Inflammation, Respiratory Viral Infection, Alveolar Sacs, Spatiotemporal Dynamics, SARS-CoV-2, Long COVID, CT Imaging, Viral and Inflammation Diffusion

## Abstract

Inflammation and lung damage in response to respiratory viral infection is a major cause of morbidity and mortality. How specialized lung alveolar structures contribute to variation in inflammatory lung damage observed in patients is a gap in current knowledge. Filling this gap is important for understanding how respiratory infections can lead to persistent and chronic sequelae after acute viral infection, including post-acute sequelae of COVID-19, or “long COVID”. Few computational models have incorporated the spatial complexity of alveolar sacs, key sites where infection and inflammation damage lung function. We propose a novel computational model, SIMALI, which represents a sample of the lung’s alveolar space as a structured 3D lattice of alveoli composed of air and epithelial cells surrounded by structural lung tissue through which virus and inflammation diffuse. SIMALI extends a previous agent-based model by adding key structural components of the lung, including physiological percentages of infectable cells and differential diffusion of virus through air and lung tissue. SIMALI’s simulation predictions are validated against the spatial-temporal growth of lung lesions from Computed Tomography (CT) scans of patients with SARS-CoV-2 infection. By combining parameters validated in a prior study with alveolar structure, the model accurately predicts the typical growth of lung inflammation observed in patient CT scans. SIMALI demonstrates how the spatial architecture of alveolar sacs and the distribution of infectable cell types in the lung constrain the spread of virus and inflammation. Furthermore, SIMALI simulations show how the initial deposition of foci of viral infection distributed across alveolar sacs is an important mechanistic cause of variation in lung damage due to inflammation. The spatial SIMALI model demonstrates a key role for the structure of the alveolar space in driving inflammatory responses. Lung alveolar structure, combined with variation in immune response and the amount and location of initial viral deposition in the lung, all contribute to the highly variable damage to lung recapitulating variation observed across patients with SARS-CoV-2 infection.

## 1 Introduction

The lung is a complex organ with multiple distinct and critical functions, including gas exchange and immune defense. Gas exchange in the lung occurs in specialized architectural structures called alveolar sacs, which include alveolar epithelial cell types I and II [1, 2]. Type I pneumocytes facilitate gas diffusion across the alveolar membrane, while Type II pneumocytes produce surfactant to maintain alveolar stability and serve as progenitor cells for tissue repair [2]. Structurally, respiration begins with the nasal passages leading to the trachea, larger branching bronchi, and increasingly small bronchiole branches ending in alveolar sacs [3]. This specialized branching structure is a key feature of lung physiology, and the large collective surface area of the alveoli (estimated at 70–80 *m*^2^ in adults) is essential for efficient gas exchange [4]. The association between alveolar epithelium and the pulmonary capillary network implies that any disruption to this architecture, whether from infection, inflammation, or injury, can rapidly compromise respiratory function.

The respiratory tract, particularly the alveolar space, is the largest permeable surface in the human body and the source of many infections, including viral, bacterial, and fungal infections. Importantly, respiratory viral infections, including influenza, respiratory syncytial virus (RSV), and SARS-CoV-2, have caused significant morbidity and mortality, while more rare but severe respiratory viral illnesses such as hantavirus remain a significant threat. Many computational models have been developed to study aspects of respiratory viral infections including those that reproduce viral dynamics [5–8], epidemiological disease spread [9, 10], and immune response to viral infection [11, 12]. Most models use Ordinary Differential Equations (ODE), often for studying viral replication, and many ODE models faithfully reproduce viral load dynamics [13, 14]. However, ODE models typically treat the lung as a well-mixed environment, which may not capture realistic infection dynamics through space and time [15], particularly for heterogeneous lung infection [16]. Non-spatial ODEs do not reflect biophysical mechanisms like lesion shape, size, distribution, and viral transport, which affect clinical outcomes of patients [17]. And, viral load alone may not reflect the true extent of lung tissue damage [18]. We previously developed the Spatial Immune Model of Coronavirus, or SIMCoV [19, 20] to address this gap by explicitly representing the spatial dynamics of viral spread and immune cell movement. While SIMCoV was parameterized using the rich datasets collected from SARS-CoV-2 patients, its spatial aspects apply to any viral infection.

SIMCoV and other agent-based models (ABMs) incorporate some spatial features of viral infection. However, most computational models of respiratory viral infection ignore spatial lung architecture and the unique cellular interactions that contribute to respiratory infection. Earlier spatial models have made important contributions by addressing distinct aspects of respiratory infection, but they did not include the full alveolar context. Levin et al. develop a spatial ABM focused on T cell search efficiency in influenza infected lung and provided grounds for SIMCoV, but do not model viral diffusion across tissue compartments [21]. Sego et al. [17] propose a modular multiscale platform for viral infection and immune response dynamics in a generic epithelial tissue model without incorporating specific lung features, and Bouchnita et al. [22] develop a hybrid multiscale model that integrated intracellular tissue, and host-level dynamics of SARS-CoV-2 and interferon response but lacking alveolar sac structure. Lukas et al. [23] develop a multimodal, tissue-level modeling approach that combines spatial imaging and neural network-based measurements to quantify viral and immune kinetics within human airway epithelium in response to SARS-CoV-2. However, none of these models address alveolar structural organization and lung-specific cell type heterogeneity. Experimental spatial studies like Desai et al. [18] and Rendeiro et al. [24] describe the spatial heterogeneity of SARS-CoV-2 infection and immune responses in autopsy tissue, though none of these experimental studies include a computational model of infection dynamics. These works collectively establish the need for spatial representation in modeling respiratory infection and provide important biological motivation for the spatial framework underpinning our current work.

Computed Tomography or CT scans of patients with respiratory infection show significant spatial heterogeneity of infection with localized and patchy lesion formation, which likely contributes to disease severity [25, 26]. CT scans are valuable tools for assessing and studying infection [18, 27–29], and longitudinal CT scans over time provide a way to validate computational predictions about spatial spread of lung damage. In particular, lung CT scans from patients with SARS-CoV-2 infection are characterized by multi-focal distribution of lesions, particularly Ground Glass Opacities (GGOs) [30, 31] and consolidations [32], both of which are likely to indicate tissue damage caused by inflammatory cell infiltration [33, 34]. This persistent tissue damage may lead to pathologies linked to long COVID or post-acute sequelae of COVID (PASC) [35], including long-term effects such as fibrotic interstitial lung disease [36–38], small airway disease (SAD) [39], and organizing pneumonia [40–42]. Thus, a computational model that can represent lung architecture and spatial features of respiratory infection can aide understanding of fundamental mechanisms leading to lung damage from respiratory infections.

In this study, we develop a computational model which includes the spatial organization of alveolar sacs called “Spatial Immune Model of Alveolar Lung Infection” (SIMALI). SIMALI introduces multiple biologically distinct compartments and cell types relevant for respiratory viral infection, i.e., air spaces, lung tissue, non-infectable (Type I) and infectable (Type II) alveolar epithelial cells. A key contribution of SIMALI is the assignment of specific diffusion coefficients, reflecting the physical properties of each lung compartment: air facilitates rapid viral diffusion, epithelial layers use previously validated epithelial cell diffusion rates, and dense lung tissues restrict inflammatory spread. We use SARS-CoV-2 data as a test case for SIMALI, demonstrating that SIMALI can reproduce the growth of spatially constrained, patchy lesion patterns that are characteristic of CT images of SARS-CoV-2 patients. The motivation behind SIMALI is not to replace ODE models, but to complement them with a spatial lens. By explicitly representing the alveolar sac geometry as a 3D lattice, SIMALI provides a tool to simulate how the specialized architecture of the lung contributes to the spatial spread of respiratory viral infections, leading to inflammatory cell infiltration and lung damage.

## 2 Results

In the following, we first describe the SIMALI modeling framework (Section 2.1) for studying the growth and spatial spread of virus and inflammation that lead to lung damage. We then describe results of SIMALI simulation experiments: how alveolar sacs constrain spatial growth of infection and resulting damage (Section 2.2); how the initial locations of infection interact with alveolar structure to determine the spatial extent of lung damage with minimal impact viral load (Section 2.3); use of sequential CT scans to analyze damage in patients (Section 2.4); replication of both patient-specific (Section 2.5) and generic (Section 2.6) patterns in the growth of lung lesions; how viral deposition and spread in alveolar sacs contributes to inter-patient variability in lung damage (Section 2.7).

### 2.1. Model overview

Viral infections often begin in the upper respiratory tract and are usually self limiting in the upper airways [44–46]. From this initial upper-airway site, viral infections can progress into the lower respiratory tract and reach the alveolar sacs [47, 48]. Viral infection of the lower airways and alveolar sacs is often associated with more severe disease [49]. The potential for disease severity and the large surface area of alveolar sacs guide our focus on alveolar sacs as the key structures in SIMALI.

**Fig. 1:**
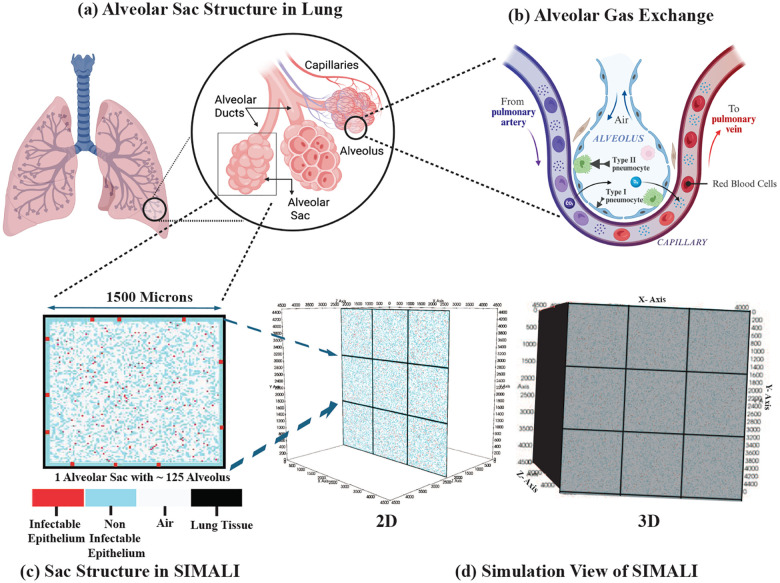
Alveolar sac structures in SIMALI. (a) shows the structure of alveolar sacs in lung. Each sac resembles a bunch of grape-like alveoli with air surrounded by alveolar epithelial cells. The sac is surrounded by capillary networks. Air flows among alveoli within a sac which is connected to an alveolar duct. (b) illustrates alveolar gas exchange, where oxygen diffuses from the alveolar sac through the cube-shaped non-infectable Type I pneumocytes into the capillary. Flat infectable Type II epithelial cells are also highlighted. (c) 2D slice of the simulated structure of a single alveolar sac containing four cell types: an outer lung-tissue layer (black), an alveolar epithelial layer composed of 95% Type I non-infectable (blue) and 5% type II infectable (red) epithelial cells. The interior of the sac contains the distribution of air and epithelial cells representing ≈ 125 individual alveoli surrounded by lung tissue. Each sac is ≈ 1500μm (100 voxels) in diameter. (d) On the left is a 2D slice through 9 sacs, and on the right 3D view of 27 sacs in the simulation grid (300 × 300 × 300 voxels; 4500μm per side, ≈ 0.1cm^3^). (a) and (b) were created using Biorender [43].

Alveolar sacs are the site of gas exchange in the lung [1](Figure 1a). Each alveolar sac consists of multiple alveoli [50, 51] which are interconnected and surrounded by a network of capillaries enabling gas exchange. As shown in Figure 1b, oxygen from inhaled air diffuses through the walls of the alveolar sacs and into the blood in the capillaries [1].

SIMALI extends the discrete cartesian grid in SIMCoV[19] to represent the alveolar region of the lung. SIMALI explicitly includes spatial features of alveolar sacs and distinct lung cell types not previously accounted for in SIMCoV. Each voxel in SIMALI is assigned one of four cell types: air, noninfectable and infectable epithelial cells, and lung tissue (space between two sacs) with corresponding diffusion rates summarized in Table 1. Unlike SIMCoV, SIMALI allows infection only in type II infectable cells [1, 2]. Diffusion of virus and inflammation varies by cell type. Diffusion coefficients range from 0 to 1 and are assigned based on the density of that voxel type. Diffusion in infectable and non-infectable epithelial cells is unchanged from the earlier SIMCoV model [19]. Diffusion through airspaces is assigned the highest rate (1), while diffusion through lung tissue is estimated as 1 × 10^−4^ of the air due to viscosity and cellular barriers [52, 53], reflecting the fact that molecular diffusion coefficients in air are approximately 10^4^-fold greater than in water [54]. Inflammation diffuses six times faster than virions [21], consistently maintained across all cell types, but inflammation diffusion in air maintains the maximum value of one. Inflammation is our proxy for lung damage in the SIMALI simulation.

Upon initialization, a specified number of cells are infected with virions. The number of initially infected cells, the spatial extent of those infections and the number of alveolar sacs infected are experimentally varied in later sections. Infected cells then enter an incubation phase followed by an expressing phase, producing virus and inflammatory signals, which diffuse through the tissue. After a delay, activated *CD*8^+^ cytotoxic T cells extravasate in locations where the inflammatory signal exceeds a threshold. These T cells randomly migrate and induce apoptosis in virally incubating or expressing cells, eventually clearing the infection. The full set of model components and their interactions is illustrated in Figure 1 of [19], reprinted as Figure A2.

Following alveolar microanatomy, each cell diameter is changed from 5μm in SIMCoVto 15μm in SIMALI to represent the size of pneumocytes [4, 55, 56] resulting in parameter adjustments by a factor of 3. The updated key parameters are summarized in Table 2. Therefore, voxels are 15μm apart, enabling aggregation over multiple cells while preserving granularity to distinguish functional cell compartments. We model alveolar sacs as cubes for ease of implementation (Figure 1c). Every sac is 100 units (1500μm) per side. Each sac is surrounded by layers of lung tissue. The outer layer of each alveolus has two types of epithelial cells: non-infectable Type I in blue and infectable Type II in red. The alveoli within a single sac are interconnected so that air flows among them. Therefore, we model the inside of the sac as a uniform random distribution of air (no color) and epithelial cells, proportioned based on an estimated 125 alveoli per sac. Each alveolus is 300μm in diameter [4]. Figure 1d shows a 2D slice of the simulation with 9 alveolar sacs and a 3D view of 27 sacs.

### 2.2 Alveolar composition and structure limit the spread of virus and inflammation

**Fig. 2:**
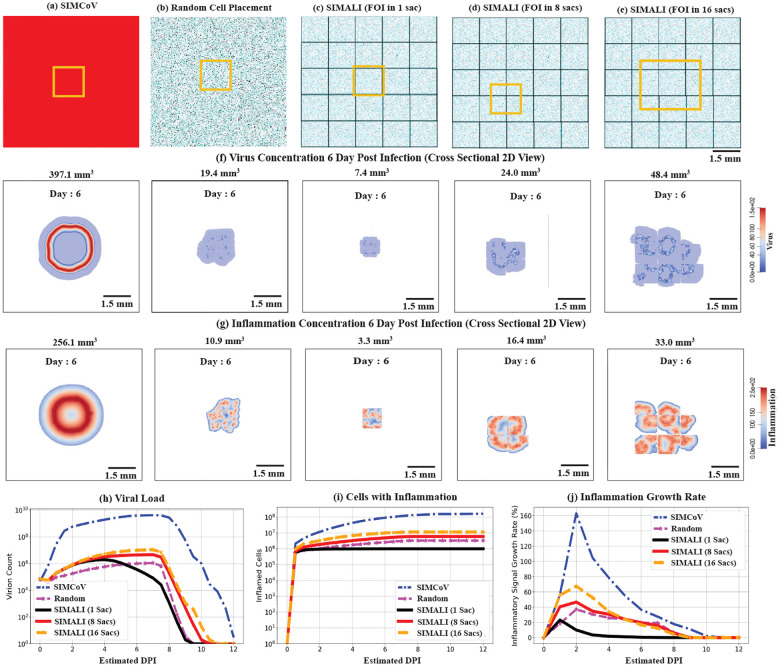
Alveolar structure and infection deposition shapes spatial dynamics of lung damage. The top row shows five simulation configurations: (a) SIMCoV: a solid 3D block of infectable cells; (b) A 3D block with a random distribution of four cell types; (c-e) SIMALI representing four cells types arranged in alveolar sacs with foci of infection (FOI) seeded across (c) 1, (d) 8, and (e) 16 alveolar sacs. All simulations start with the same number of FOI and identical virion counts, placed in the yellow box. Cross-sectional 2D views at 6 DPI show the concentrations of virus (f) and inflammation (g) across all five configurations. The corresponding volume of cells containing virus and inflammation is also indicated. All simulations except SIMCoV (with 1000 × 1000 × 1000 voxel ≈ 3.4*cm*^3^) use a 600 × 600 × 600 voxel domain ≈ 0.7*cm*^3^ and run for 12 DPI with T cell arrival at 7 DPI. SIMALI produces spatially heterogeneous, structured lesions; in contrast, SIMCoV produces a homogeneous ring of infection and inflammation. The random configuration shows patchy distributions of virus and inflammation that are unconstrained by dense lung tissue. (h–j) show temporal dynamics of viral load (h), inflammatory cell count (i), and inflammation growth rate (j). SIMCoV produces the highest value for viral load, cells with inflammation, and inflammation growth rate due to its all-infectable, unstructured framework, while SIMALI configurations consistently reduce all three metrics, with the amount of damage determined by the number of alveolar sacs seeded by the initial placement of FOI. Video demonstration is available here.

In order to understand how alveolar structure and cell distribution affect spread of virus and inflammation, we first compare SIMALI (Figure 2c) to a homogeneous, uniform, solid 3D block of infectable cells (the previous SIMCoV model Figure 2a) and then to a 3D block with the four cell types from Table 1 placed at uniform random positions. We initialize all three simulations 2(a–c) with the same diffusion parameters for each cell type and the same number of initially infected cells within the same volume (outlined in yellow boxes) which we consider the focus of infection (FOI). In all experiments T cells arrive at 7 days post infection (DPI).

What differs between experiments is the composition and structure of the cell types: panel a shows the original SIMCoV - a 3D block consisting only of infectable epithelial cells; panel b introduces the 4 cell types used in SIMALI, placed at random, with corresponding diffusion rates from Table 1; panel c introduces the alveolar structure of SIMALI. In each of these 3 cases (panels a-c) the same number of cells are infected in the same yellow cube that is the size of a single alveolar sac. We then compare across the three simulations to answer the question: if virus is inhaled into a single alveolar sac, how do the cellular composition and structure of the sac affect the spread of virus and inflammation?

Spatial spread of virus (Figure 2f) and inflammation (Figure 2g) are shown at 6 DPI vertically below corresponding simulated structures. The simulations are shown in video demonstration, The volume of cells containing viral infection or inflammation at 6 DPI are shown at the top of each panel. In SIMCoV (Figure 2a), viral spread and inflammation grow in an expanding uniform ring (Figure 2f, g, first column) which fills a large volume (397 mm^3^ of virally infected cells and 256 mm^3^ of cells with inflammation) by 6 DPI. When the four cell types with variable diffusion are introduced in panel b, the spread is more heterogeneous and reduced to 19.4 and 10.9 mm^3^, 3–5% of the volume of the SIMCoV simulation. When we introduce alveolar structure in panel c, the volume of infected and inflamed cells are further reduced to 7.4 and 3.3 mm^3^, respectively, 1 – 3% of the SIMCoV simulation. These experiments show that introducing a physiologically realistic mix of air, infectable and non-infectable epithelial cells, and lung tissue dramatically change the extent and spatial pattern of spread of infection and inflammation. Further, the specific architecture of alveolar sacs surrounded by lung tissue that slows diffusion among alveolar sacs constrains spread to within the originally infected sac at 6 DPI before CD8+ T cells arrive in the lung. This suggests that the alveolar structure can limit the spread of damage from inflammation to 1% of a simpler model considering only infectable cells.

Additional effects of the composition and structure of the alveolar space are evident in panels h, i and j. Panel h shows SIMCoV(blue dashed line) has by far the highest viral load, the random distribution of four cell types (magenta dashed line) has the lowest early viral load, but it continues to grow until T cells arrive, while SIMALI (black line) peaks earlier at 4 DPI. SIMCoV also has a much larger number of cells that contain inflammation (panel i) and by far the hightest growth rate of inflammation (panel j), while SIMALI has the lowest growth of inflammation. Collectively, these results establish that the composition and structure of alveolar sacs shape viral and inflammation dynamics and substantially limit their spread.

### 2.3 Location of initial viral infection within alveolar sacs affects the spatial spread of virus and inflammation

Here we address the question of how the placement of initial FOI across multiple alveolar sacs changes the spread of virus and inflammation. All SIMALI simulations are initialized with the same number of initially infected cells. In panels c and d, the initially infected cells are placed in FOI with the same volume (yellow cube) constrained to one alveolar sac in (c) and crossing eight sacs in (d). In panel (e) we double the radius of the FOI so it is eight times larger and crosses 16 sacs with the same number but a lower density of initially infected cells.

In Figure 2f and g, the columns below 2c, d, and e show virus and inflammation spread at 6 DPI. The infection remains contained in whatever number of sacs are initially infected (one in panel c, eight in panel d and 16 in panel e), although the sacs are not completely filled in the 8 and 16 sac cases which have 5-fold and 10-fold greater volume of inflamed cells compared to the 1 sac case. The dynamics of viral load and inflammation increase are similar for 1, 8, and 16 sacs configurations, with increasing virus and inflammation with more initially-infected sacs. However, when only 1 sac is infected, the viral load begins to decrease after 4 DPI, while in the configurations with more sacs infected, there is an increase in viral load until 7 DPI when T cells arrive (Figure 2h, compare 1 sac-black line, 8 sacs-red line, 16 sacs-dashed yellow line).

SIMALI is designed to identify cells that have been exposed to inflammation as a measure of lung damage, and we assume that 12 DPI are not long enough to account for cell recovery from inflammatory damage. This is consistent with observations in some patients [57–61]. These results show that even with identical initial number of virions infecting an individual, if more alveolar sacs are infected, inflammation spreads more rapidly and affects more of the lung, potentially increasing lung infection and reducing lung function through alveolar sac involvement. The scaling of inflammation with the number of initially seeded sacs demonstrates that the spatial distribution of infection foci, and not just the initial viral load, is a primary determinant of inflammation in the lung in the context of viral infection.

A comparison of the inflammation growth rate (%) over time provides the clearest illustration of the effect of alveolar sac structure and the rationale for SIMALI’s design. A uniform sheet of infectable epithelial cells shows the fastest early growth of inflammation, peaking at approximately 160% around 2 DPI (Figure 2j-blue dotted line). By comparison, the peak growth rates are approximately 22% for 1 sac (Figure 2j-black), 47% for 8 sacs (Figure 2j-red), and 68% for 16 sacs (Figure 2j-yellow dashed). These results show that accounting for the composition and structure of alveolar sacs limits the growth of inflammation.

In the supplement (simulation video), we further test how the distribution of cell types affect the spread of virus and inflammation in two other scenarios in the 8-sac model: 100% epithelial cells with only 5% infectable cells (no air or lung tissue in, Figure A1c), and 5% infectable cells in air (Figure A1d). As expected, the absence of air strongly reduces viral and inflammation growth, and the absence of lung tissue allows unconstrained growth.

Holistically, Figure 2h–j demonstrate that alveolar sac structure suppresses viral load, inflammation, and daily growth of inflammation in a manner that scales with the spatial extent of initial viral deposition. Random cell placement, despite having the same four cell types and the same percentage of infectable cells, shows lower viral spread and inflammation values than we observe in SIMALI with multiple-sac configurations. This demonstrates that the spatial organization of the lung into multiple alveolar sacs can limit viral load but depending on the initial deposition of virus may either decrease or increase the amount of lung that is inflamed. SIMALI highlights three factors essential for accurately modeling spatial viral and inflammation dynamics: (1) the presence of distinct alveolar cell types with heterogeneous diffusion properties, (2) the structured spatial arrangement of the cells, and (3) the location of initial virion deposition.

### 2.4 Estimating lung damage in patients

We test the SIMALI model predictions using Computed Tomography (CT) imaging of lungs from patients infected with SARS-CoV-2 [26], which provides a longitudinal dataset of CT scans of patients post symptom onset. We use the estimated day of infection (DPI) from the original study to determine DPI. In these comparisons, we assume that the growth of damaged lung tissue in CT scans is a reasonable test of SIMALI predictions for the spatial growth of inflammation that damages the alveolar space. Thus, we quantitatively compare the growth of individual lung lesions visible in the CT scans to the growth of inflamed regions predicted by SIMALI. Opaque areas in lung CTs indicate inflammatory cellular and fluid infiltration resulting from lung infection [33, 34]. We quantify the spatiotemporal dynamics of inflammationinduced lesions from patients with COVID-19 in this dataset and compare them quantitatively with predictions of lung inflammation from SIMALI.

**Fig. 3:**
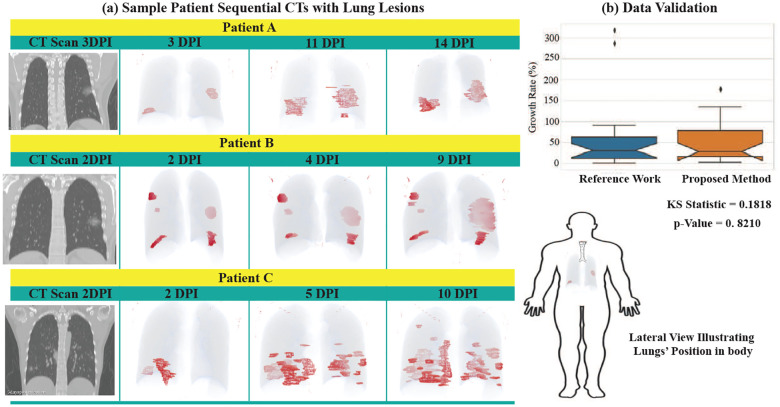
Sample patient CT lesion visualization and validation. (a) Sequential CT scans for three sample patients from data source [26], showing 2D CT slices (leftmost) and 3D lesion progression over estimated DPIs, with lung tissue in gray and lesion damage in red (coronal view). A human figure contextualizes lung positioning. Lesion growth rates vary across patients, reflecting heterogeneous infection dynamics. (b) Notched box plots comparing the total growth rate across all lesions between the original source [26] and the summing of individual lesion growth rates we calculate here. The central notch, box width, and whiskers represent the median, interquartile range, and data range, respectively, with outliers shown individually. A KS statistic of 0.1818 and p-value of 0.8210 [62] confirm no significant difference between the two distributions, supporting that our individual lesion calculations are consistent with cumulative lesion calculations in the original publication [26].

This lung CT dataset includes 29 COVID-19 positive individuals (mild to moderate) with serial CT scans over an average of 50 days. We identify 20 patients, each with 2–3 time points showing positive lesion growth over the first 10 DPI. We construct 3D visualizations of the scans to observe the spatial progression of each lesion over time. Figure 3a shows three representative patients (A, B, C). The leftmost panels show 2D slices that provide a view of the internal lung structure. The subsequent panels show tissue damage highlighted in red, which indicate opaque regions in CTs on the gray background of lung tissue. Between 2 and 14 DPI, most lesions show asymmetric and increasing volume, with some smaller lesions merging into larger lesions (Figure 3a, patient C). Most lesions are observable in the first CT available for a patient, then grow in later DPIs. In a few rare instances, new lesions were observed to emerge at later time points (Patient C, compare 2 and 5 DPI). All of the available lung CT images from the 20 patients display distinct temporal patterns and extents of lung damage; full 3D visualizations are available in [63]. We use these reconstructed lesion volumes to validate SIMALI. Lesion identification and procedures to quantify their growth are described in Methods. We validate our method of calculating individual lesion volume by comparing the summed total lesion volume against the source study’s radiologist calculated lesion volumes (Figure 3b-reference work [26]). While our quantification generates slightly lower growth rates with fewer outliers relative to the source study, our measured lesion volumes are statistically consistent with the radiologist calculated volumes (KS statistic 0.1818 and p-value 0.8210)[62].

**Fig. 4:**
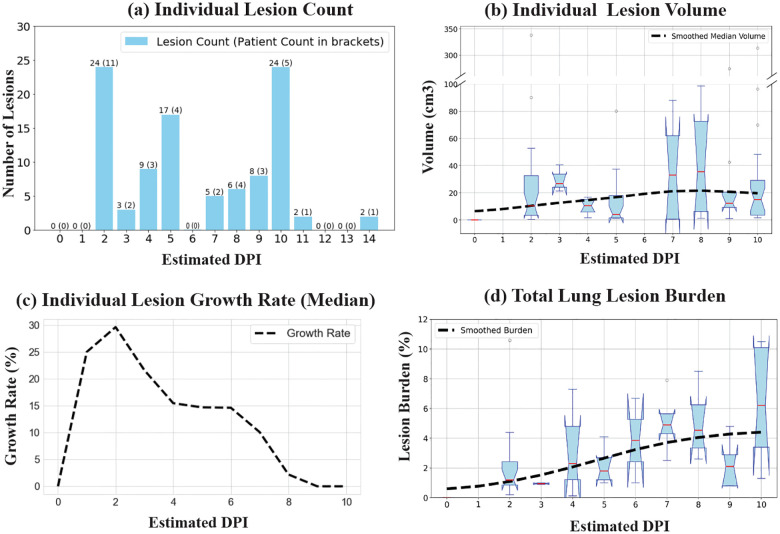
Individual and total lung lesion analysis to estimate inflammation growth rate and lung lesion burden. (a) Individual lesion count per DPI across 20 patients, with patient count in brackets. (b) Notched boxplots of individual lesion volumes (cm^3^) over estimated DPIs. The smoothed median (black dashed line) is obtained by Gaussian smoothing of interpolated median volumes, showing growth peaking at approximately 8 DPI. (c) Median individual lesion growth rate derived from the smoothed volumes in (b), peaking at ≈30% around 2 DPI before declining steadily. (d) Total Lung Lesion Burden (LLB) distributions across available DPIs for the 20 patients; Gaussian smoothing of interpolated median LLB values creates the continuous burden trajectory (black dashed line), capturing the spatial extent of inflammatory lung damage over the course of infection.

One limitation of the Kassin et al. dataset is that the indicated DPIs are estimates, derived from a combination of many studies that include patientreported symptom onset dates and temporal metadata of viral load [26]. In addition, the patients have scans at various DPIs, leaving some time points unrepresented or represented by only a single patient. This contributes to the variability in observed growth rates across the cohort. Most of our analysis focuses on the growth rates of individual lesions averaged over all patients. Only lesions that remain distinct and show reliable positive growth across multiple CT scans are included. We identify 46 unique such lesions as shown in Figure 4a and report individual lesion volumes for each DPI in Figure 4b. Given the significant variability across lesions at individual DPI in notched box plots, we use the median volume per DPI to calculate inflammation growth rate (Figure 4c) and total lung lesion burden (Figure 4d). For missing days (0, 1, 6), we interpolate and apply Gaussian smoothing to reduce noise and reveal clearer trends (see Methods). The smoothed median (dashed line) shows steady lesion volume peaking around 8 DPI, followed by a decline by 10 DPI. Figure 4c shows the smoothed median growth rate of individual lesions, peaking at ≈30% around day 2 before declining steadily, reflecting rapid early infection followed by progressive immune-driven containment. This agrees with the likelihood that viral infection peaks at 5–6 DPI followed by the T cell response, which leads to clearance by 10 DPI [46, 61].

To account for variability in opacity segmentation and noise in individual lesion volume calculation, we incorporate Lung Lesion Burden (LLB) as a complementary and more robust metric than direct lesion measurement. LLB is defined as the ratio of total lesion volume to total lung volume for CT images. LLB quantifies the extent of inflammatory tissue involvement, serving as an established prognostic indicator of disease severity in CT imaging [64, 65]. LLB also enables consistent comparison across patients, timepoints, and computational models. Figure 4d shows LLB distributions across DPI for the 20 patients from Kassin et al. [26], with notably lower variability than individual lesion volumes. Gaussian smoothing over missing timepoints generates a continuous median lesion burden trajectory (black dashed line). For comparison with simulation outputs, LLB is scaled by the ratio of the simulation domain volume to the average patient lung volume, which enables direct comparison with SIMALI predictions. We report an illustrative example in Appendix Figure A5, where patient LLB values are scaled to a simulation volume of 0.7*cm*^3^ (see Methods).

### 2.5 SIMALI can reproduce patient-specific individual lesion growth dynamics

To compare SIMALI estimated lesion volumes with lesions measured from CT images, we quantified individual lesions that are of similar size to those computed using SIMALI. Currently, SIMALI can simulate up to 7 *cm*^3^ of lung tissue (≈ 2 billion cells), well below the full lung volume of 4000–6000 *cm*^3^ shown in CT images [26, 55]. Simulating the entire lung was computationally prohibitive, so we focus on quantifying single lesions that enable direct comparisons between simulated inflammation and observed CT lesion volumes. The goal is to match both lesion volume and growth trends with minimal parameter tuning and with biologically plausible initialization.

**Fig. 5:**
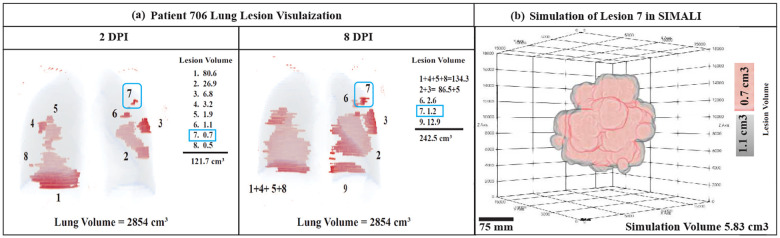
Patient-specific lesion replication with SIMALI. (a) 3D lung visualization of Patient 706 at 2 and 8 DPI, with individual lesion volumes labeled. Lesion 7 (highlighted), measuring 0.7 *cm*^3^ at 2 DPI and 1.2 *cm*^3^ at 8 DPI, remains spatially distinct across both timepoints and is selected for simulation. (b) 3D reconstruction of SIMALI simulated inflammation for Lesion 7, initialized with spatially informed parameters within a 5.83 *cm*^3^ domain. The 2 DPI inflammatory volume (0.7 *cm*^3^, red) is overlaid on the 8 DPI volume (1.1 *cm*^3^, gray), demonstrating close agreement with observed lesion growth and spatial expansion driven by cell specific diffusion through alveolar structures. Video demonstration is available here.

To validate SIMALI against lung lesions from patient CT images, we recreate the spatiotemporal progression of an individual lung lesion in SIMALI to compare with a lesion from the CT scan of Patient 706 [26]. Patient 706 has CT scans at estimated 2 and 8 DPI with confirmed positive lesion growth. Figure 5a shows 3D lung and lesion visualizations at 2 and 8 DPI for Patient 706. At 2 DPI, 8 distinct lesions are visible with a total lung lesion volume of 121.7 *cm*^3^ across a lung volume of 2854 *cm*^3^. By 8 DPI, several lesions have merged: lesions 1, 4, 5, and 8 coalescing into a single region of 134.3 *cm*^3^, and lesions 2 and 3 merging to 86.5 *cm*^3^. This brings the total lesion volume to 242.5 *cm*^3^. We selected Lesion 7 for simulation because it remained spatially distinct across both time points, growing from 0.7 *cm*^3^ at 2 DPI to 1.2 *cm*^3^ at 8 DPI, providing a clean ground truth for comparison to SIMALI without the confounding effects of lesion merging. This specific lesion grew by 71% between 2 and 8 DPI, similar to the total growth of all lesions of 80% over that same time period, suggesting that this single lesion is representative of growth in this particular patient.

Based on the relationship between sac dimensions and lesion volume calculations in SIMALI, we estimate that an initial infection in approximately 395 alveolar sacs can reproduce the observed lesion volume in Patient 706 at 2 DPI. 395 alveolar sacs corresponds to ≈ 395 million cells in the lung. Figure 5b shows the resulting 3D simulation, where the inflamed cell volume at 2 DPI (0.7 *cm*^3^, red) is overlaid on the 8 DPI volume (1.1 *cm*^3^, gray), closely approximating the observed growth from 0.7 to 1.2 *cm*^3^ in the patient. The full SIMALI simulation comprises ≈1.728 billion cells representing a 5.83 *cm*^3^ lung volume. The spatial expansion pattern of lesion growth in SIMALI is consistent with asymmetric diffusion-driven inflammation filling alveolar sacs based on the spatial positioning of initial viral deposition, creating the patchy, localized lesion morphology reminiscent of lesions observed in patient CT scans of virally infected lung (simulation demo). These results show SIMALI faithfully reproduces inflammatory lesion burden seen in patients both spatially and quantitatively.

### 2.6 SIMALI recapitulates total lung lesion spatiotemporal dynamics in patient CT scans

In this section, we compare SIMALI predictions of the growth of inflammatory damage to the growth of lesions averaged across patients. We use biologically realistic parameters validated as default parameters that matched patient viral load from SIMCoV [19]. We add the viral and inflammation diffusion parameters from Table 1. We initiate SIMALI with an FOI that is the size of a single alveolar sac (a million cells), as in Figure 2c and 2d, but we place the FOI randomly so that it may (rarely) infect only 1 sac, but likely infects up to 8 sacs. An estimated 13,000 cells in this volume are infectable Type II pneumocytes. Studies suggest that the number of inhaled SARS-CoV-2 virions required to establish an infection is likely on the order of a few hundred [66, 67], which we approximate as infecting 1% of the 13,000 infectable cells (Table A1) for the mild to moderate infections included in our dataset.

**Fig. 6:**
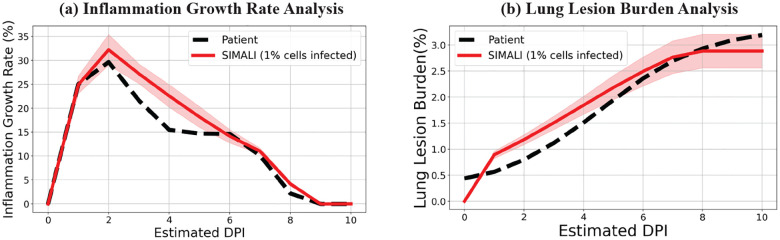
SIMALI reproduces patient trends in lung damage dynamics using default parameters. (a) Inflammation growth rate over time for SIMALI simulations with 1% (red line) initial infection levels, compared to median patient data (black dashed line). The shaded area around the red lines shows variability across 5 runs. The simulations were run on a 600 × 600 × 600 voxel space, representing a 0.7*cm*^3^ region of lung. All curves exhibit a sharp rise in early infection, peaking around 2 DPI, followed by a gradual decline. The model closely replicates the timing and magnitude of this peak, including the sharp decrease in growth rate around 7 DPI, corresponding to T cell arrival. (b) Lung lesion burden (LLB) over time for the same simulations and patient median data (scaled with simulation). The LLB increases rapidly during early infection and plateaus without significant decline, reflecting the sustained presence of inflammation.

Figure 6 compares the predicted growth of inflammation from this biologically realistic parameterization of SIMALI with the average growth of lesions from patients. It shows SIMALI results follow the median patient inflammation growth rate (a) and LLB (b). In Figure 6a, SIMALI predictions parallel patient CT scans, showing a sharp increase in the inflammation growth rate, peaking around 2 DPI and dropping until resolution at 10 DPI.

Figure 6b shows LLB in SIMALI with 1% initial infection levels compared to the median patient LLB (scaled with simulation size). Both simulation and patient curves demonstrate a rapid rise in lung lesion burden during the early phase of infection, with the steepest increase occurring around 2–4 DPI. Unlike the inflammation growth rate, LLB does not decline over time in either the simulation or patient CTs due to the continued presence of tissue damage caused by inflammation [68]. SIMALI reproduces the persistence of lung damage even as viral load clears. By simulating LLB and inflammation with realistic parameters, SIMALI provides a mechanistic explanation for the typical patient lung damage observed in patient CT scans.

To study the impact of varying initial infection foci, we perform simulations by infecting 1% to 100% of infectable cells (number of FOI is summarized in Table A1). For each FOI percentage, five random spatial locations are modeled. Due to the randomness of FOI placement, FOI clusters are distributed in more than one alveolar sac (Figure 2d scenario). This random placement of FOI introduces stochastic variation in infection distribution, analogous to spatial and viral-load variability at infection onset in individuals exposed to respiratory viral infection. Detailed analysis of the individual simulation runs ranging 1% to 100% is presented in the Appendix Figure A4. Results show faster growth with fewer FOI (1%) because less dense infections have more nearby cells to spread to within the same alveolar sacs. These simulations show that the most biologically plausible 1% infection scenario predicts the trends in patient lung damage more closely than the other scenarios. This further supports the claim that SIMALI captures the dynamics of lung damage using biologically realistic parameters.

### 2.7 Sensitivity analysis of key parameters in SIMALI captures disease variability in patients

**Fig. 7:**
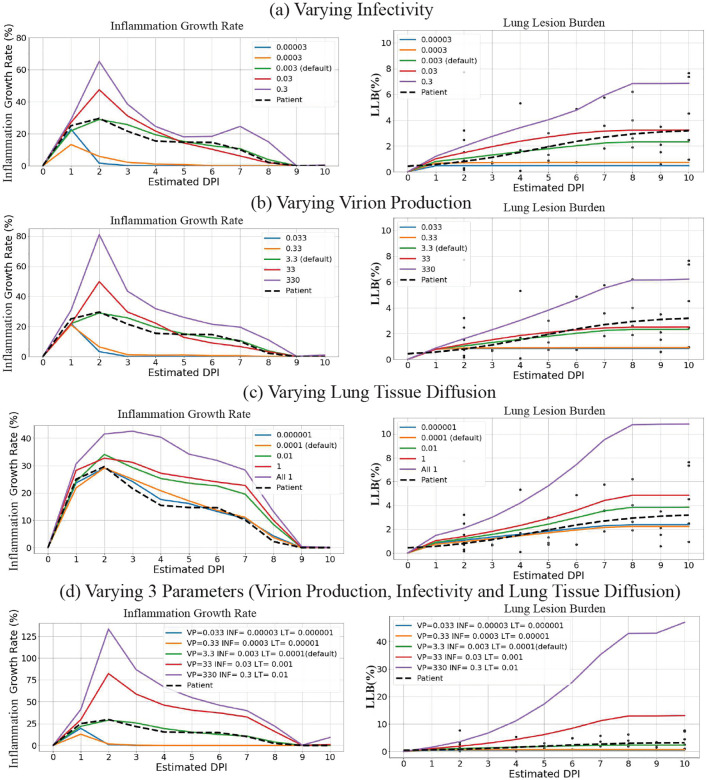
Sensitivity analysis of key model parameters: infectivity, viral production, and lung tissue diffusion account for disease variability in patients. Panels (a–d) illustrate the effects of varying biologically relevant parameters on inflammation growth rate (left column) and lung lesion burden (LLB, right column) in SIMALI. Each parameter is shown in color compared to the median patient in dotted black. Default parameters used in SIMALI are indicated. (a) Varying infectivity shows that lower infectivity results in slower inflammation growth and reduced LLB. (b) Varying virion production reflects control of viral replication in cells. Viral suppression leads to significantly lower inflammation and damage. In contrast, higher production of the virus results in severity in infection growth and burden. (c) Varying lung tissue diffusion demonstrates how reduced diffusivity associated with dense tissue limits the spatial spread of infection and lesion progression. (d) Simultaneous variation of infectivity (INF), virion production (VP), and lung tissue (LT) diffusion captures a broad spectrum of outcomes, highlighting that default parameters (green line) capture the median patient scenario (dashed black line), and variability of aforementioned parameters explains inter-patient disease severity differences.

Analysis of CT scans of patients in Section 2.4 reveal notable variability in lesion growth and burden. Patients also experience significant variability in damage to lung function. To assess the ability of SIMALI to model this variability seen in patient outcomes, we perform a sensitivity analysis on key parameters governing infection dynamics in SIMALI: viral infectivity (Figure 7a), virion production rate (Figure 7b), and lung tissue diffusion coefficients (Figure 7c). SIMALI incorporates crucial differences from previous models, and sensitivity analysis tests how the important new parameters impact lung inflammation.

Sensitivity analysis shows that both inflammation growth rate (Figure 7, left column) and LLB (Figure 7, right column) are sensitive to all three parameters: infectivity (Figure 7a), virion production (Figure 7b), and lung diffusion (Figure 7c). Figure 7a shows that higher infectivity leads to rapid and widespread infection, resulting in steeper and higher inflammation growth rate and LLB. Conversely, lower infectivity leads to slower lesion growth and reduced overall lesion burden (Figure 7a), likely resulting in less tissue damage. Figure 7b presents the effect of changing the virion production rate in virally expressing cells. Higher virion production amplifies epithelial cell infection and the inflammatory response, driving up both inflammation growth rate and LLB while lower virion production reduces inflammatory growth and overall LLB.

Figure 7c explores how changes in the diffusion rate of virions and inflammation through lung tissue affect the progression of infection. In sections 2.2 and 2.3, we describe the effects of multiple alveolar cell types, each with varying diffusion rates, in the SIMALI model and show that they contribute to the containment of lung lesion progression. The purple line in the plot shows the case in which all diffusion rates are the same (1), leading to maximal inflammation. Lung tissue is modeled as denser than air and epithelial cells, and thus diffusion is lowest (0.0001), impeded by the extracellular matrix which limits the mobility of viral particles and signaling molecules [69, 70]. These results show that in SIMALI, higher lung tissue diffusion increases both inflammation growth rate and LLB. Comparing Figure 7a, b, and c, the sensitivity of lung viral diffusion is lower than that of infectivity and virion production. Lower diffusion restricts spatial spread, reducing lesion burden, but paradoxically elevates the inflammation growth rate. These findings are consistent with biological observations, where reduced diffusivity in dense regions limits viral propagation but may also contribute to inefficient immune signaling, potentially leading to prolonged local inflammation. Poor cytokine diffusion in structured or dense tissue can also limit immune signal range, potentially reducing overall infection clearance [71, 72].

We set default parameters for SIMALI (green line) to biologically plausible parameters: i) those previously validated in SIMCoV matching viral dynamics in a different patient study, ii) diffusion parameters reflecting the density of air and lung tissue (Table 1) and iii) assuming 1% of infectable cells are initially infected, consistent with prior studies. These assumptions are consistent with observed inflammatory growth and LLB in the median patient data (Figure 7, dotted black lines). For each parameter, the default inflammation growth and LLB lie in the middle of the range of infectivity, virion production, and diffusion. The close fit of the biologically realistic default parameterization to the patient data is particularly clear in Figure 7d. The default parameterization (green), closely tracks the median patient (black). The combined effects of simultaneously varying all three sensitive biologically significant parameters (virion production, infectivity, and lung tissue diffusion) track the observed wide variation in individual patients. By variying all three parameters, we see even more clearly that the SIMALI parameters (green line-default) closely track the median growth rate of damage in patients (dashed black line).

## 3 Discussion

### 3.1 Summary

Respiratory viral infections including influenza, SARS-CoV-2, and other coronaviruses cause morbidity and mortality not necessarily from the viral infection itself, but also from the sustained lung inflammation and tissue damage that follow acute infection; e.g diffuse alveolar damage, inflammatory cell infiltration, and fibrin deposition observed post-mortem [73, 74]. Understanding how this inflammatory damage initiates, spreads, and varies across patients requires a modeling framework that captures the spatial dynamics of the lung. Many computational models including ODE based mathematical models include key features of respiratory viral infections such as viral dynamics, interferon control, and immune response to infection. However, ODE models that treat the lung as a well-mixed environment cannot provide insight into how heterogeneous structural features of the lung affect the spread of viral infection and inflammation. SIMALI provides the structural modeling framework to better understand how alveolar lung architecture impacts viral dynamics and inflammation in the lung.

A primary finding of this work is that alveolar sac structure is a key determinant leading to spatially heterogeneous, patchy lung lesion formation consistent with the images of lung lesions from patient CT scans with Ground Glass Opacities and consolidations [30–32]. Because disease severity arises not necessarily directly from the viral infection itself but from the sustained lung inflammation and endothelial damage that follow [75, 76], SIMALI is the first model to directly assess the inflammation in the lung to connect modeling results with patient CT data.

Validation of SIMALI predictions against longitudinal CT data from 20 COVID-19 patients confirms that SIMALI captures the key phases of disease progression using two complementary metrics: inflammation growth rate and lung lesion burden (LLB). Inflammation growth rate peaks sharply around 2 DPI and declines steadily starting at 7 DPI leading to clearance by 10 DPI, likely driven by T cell arrival at 7 DPI. LLB rises rapidly in early infection and plateaus without significant decline, reflecting rapid inflammatory cell infiltration after infection and sustained inflammation which can induce tissue damage that persists beyond viral clearance. Quantitative CT studies demonstrate that central–peripheral distributions of GGOs and consolidations correlate with clinical outcomes [27]. SIMALI reproduces both early expansion and sustained damage patterns seen in lungs of patients, including replicating patient-specific lesion growth. These results support the use of SIMALI as a potential predictive tool for assessing potential individual patient outcomes [77].

SIMALI offers multiple insights into SARS-CoV-2 infection and immune dynamics within the lung. A primary finding is that the spatial structure of the lung, particularly the alveolar sac structure, limits viral spread and contains damage caused by inflammation. Unlike the simpler SIMCoV model and other models that assume homogeneous tissue including homogeneous infection and diffusion across lung, SIMALI simulates air space and different lung cell types that act together to constrain the spread of virus and inflammation (Figure 2). There has been limited work on how spatial relationships impact SARS-CoV-2 infection and damage propagation within the lung. Previous agent-based modeling SIMCoV[19] and multiscale models [22] reveal that the spatial dispersion of initial foci and interferon–virus interactions strongly influence infection dynamics. Spatially resolved pathology using imaging mass cytometry and spatial transcriptomics reveals how regional immune–epithelial cell organization and patchy diffuse alveolar damage within the lung microenvironment contribute to disease severity and progression [24, 78, 79]. SIMALI suggests that viral infection in the spatially heterogeneous architecture of alveolar sacs lead to patchy and discontinuous lesions commonly observed in patient CT scans (Figure 3a), offering a mechanistic explanation for clinical observations. By coupling spatial dynamics with validated viral and immune parameters, SIMALI reproduces patient-specific lesion growth rates and lung lesion burden over time.

SIMALI enables us to test how the same number of viral particles infecting different spatial compartments in the lung affect lung inflammation and lesion burden. We tested the initial number of virions required to initiate a sustained infection. Simulations suggest an initial infection count on the order of 100–1000 virions, approximately 1% of total infectable cells in an alveolar sac, lead to sustained infection. Multiple studies support this as a realistic estimate with the number of inhaled SARS-CoV-2 virions required to establish an infection [66, 67]. Reproducing the patient specific lesion example demonstrates that SIMALI, when initialized with a structurally informed infection site and run with biologically validated parameters, can accurately replicate the observed trajectory of an individual lesion in the lung of an individual patient. This supports the model’s capability to simulate patient-specific outcomes and highlights its potential to evolve into a predictive tool for individual outcomes with increasing involvement of more alveolar sacs. Current limits on SIMALI computation allows us to test lesion burdens in a small part of the lung, with expected ranges of inflammation growth rate and LLB of up to 3%. We expect that if viral infection occurs in more alveolar sacs, the inflammatory damage and lesion burden can significantly expand, leading to the long term inflammatory damage reported in some patients with post-viral sequelae. Our initial SIMALI simulation example forms the basis for investigating the mechanisms that drive inter-patient variability in individual lesion progression. Extension of SIMALI into larger areas of the lung can provide projections of disease trajectories and ultimately different outcomes for patients.

Sensitivity analysis highlights viral infectivity and virion production rate as the most influential parameters driving inflammatory growth and LLB. Lung tissue diffusion plays a secondary but biologically meaningful role. Together, these three parameters recapitulate distinct facets of the host interferon (IFN) response, particularly type I and III IFNs which reduce infectivity by suppressing viral replication and upregulating interferon-stimulated genes to interfere with viral synthesis, protein translation, and reducing virions released as well as downregulating viral entry receptors (like ACE2 for SARS-CoV-2) and enhancing the expression of antiviral restriction factors at the epithelial surface [80–84]. These outcomes are consistent with experimental studies showing that early and robust IFN signaling correlates with lower viral loads and reduced lung damage, whereas delayed or deficient IFN responses lead to uncontrolled viral replication and severe disease progression [85–87]. Suppression of viral entry into cells, suppression of intracellular viral replication, and restriction of inflammatory signal diffusion through tissue reflect effective IFN responses which constrain infection spread and tissue damage. In contrast, high values of all three mimic impaired or delayed IFN signaling that produce rapid viral spread and high lesion burden consistent with severe COVID-19 [85–87]. Simultaneously varying all three parameters spans a broad spectrum of disease severity that maps onto the inter-patient variability observed in clinical data, emphasizing that spatial containment enforced by the alveolar sac architecture and early immune modulation jointly govern the progression of viral infection induced lung damage.

### 3.2 Caveats, limitations and future work

While SIMALI presents a significant advance in modeling respiratory infection with the inclusion of spatial lung architecture, SIMALI has one major inherent limitation in the ability to model the full human lung. The current the model represents billions of individual cells, but current simulation capabilities do not extend to modeling the entire human lung volume (4000 to 6000 cm^3^) which is significantly larger than the SIMALI modeling volume (7 cm^3^). The current SIMALI simulation size limits the direct comparisons of absolute lesion volumes to localized regions, in particular how overall lung function might be impacted with infection in more alveolar sacs leading to larger lesions. In the current simulations, all lesions grow uniformly, unlike patient cases where multiple lesions exhibit distinct growth rates. Thus, multi-lesion scenarios are not simulated, and individual lesions are modeled separately. The scenario of two lesions merging is not included in the experiments. The precise number of alveolar sacs initially infected to form an observed lesion remains an important open question for future work. In the current framework, lesion formation depends on the number of initially infected sacs, while propagation across sac boundaries is limited by diffusion constraints. Whether clinically observed lesions arise primarily from initial viral deposition or from subsequent spread through sacs remains to be determined.

An additional limitation of SIMALI is the actual physical representation of the alveolar sac. For implementation simplicity, alveolar sacs are currently modeled as cube-like rather than sphere-like structures. Furthermore, our patient cohort reflects mild to moderate cases; the severe case scenarios are not covered which may show different levels of lesion burden and inflammation growth rates. Information on patient-specific innate immune effects, which vary with patient age and medical history, was unavailable for this study. Processes such as cell repair, fibrosis, and long-term remodeling remain absent from the model but are crucial for predicting recovery trajectories. Addressing these gaps will require not only computational advances but also integration of longitudinal imaging and immunological datasets.

To further refine and validate the SIMALI model, future work will focus on creating individual patient scenarios based on CT scans and incorporating laboratory results. This integration of different data types will be crucial for personalized modeling of disease progression. CT scans show lesions frequently appear on the periphery of the lung [26]. This presents additional hypotheses for future exploration, including whether immune responses are slower, viral spread is faster, or structural factors specifically influence viral dissemination in the lung and lesion formation in these specific lung regions. Addressing these areas will improve the biological relevance and predictive accuracy of the SIMALI model for personalized treatment strategies for respiratory viral infections including SARS-CoV-2.

## 4 Methods

### 4.1 Patient Data Analysis and Comparison Metrics

Many studies have used lung CT scans and chest X-rays to detect COVID-19 [88–91]. Most of these propose automated AI-based approaches for lung and lesion detection. Therefore, public datasets of CT scans [92–94] are available with segmented lungs and lesions. In most cases, these are not sequential scans over time. However, we have used two datasets [92, 94] with pre-segmented lungs and lesions to calculate individual lesion volumes (sample analysis is shown in Figure A3)

In order to compare the growth of patient lesions to SIMALI simulations, we use the sequential CT scan dataset from Kassin et al. [26] for multiple days before and after symptom onset. Patient demographic data is detailed in their methods section [26]. The dataset contains 739 patients with confirmed COVID-19. All were clinically considered mild to moderate cases. 29 COVID-19 positive individuals underwent serial CT and laboratory tests over an average period of 50 days. The study employs both manual and AI-based segmentation to quantify lung opacities, including ground-glass opacities (GGO)[30, 31] and consolidation [32]. The study provides important insights into the temporal onset of opacities; i.e. according to their data, lung opacities appeared approximately 3.4 days before symptom onset, with a peak occurring around the day of symptom onset [26].

From this dataset, we obtained the raw CT scans. We identify 20 patients, each with 2–3 time points showing positive lesion growth over the first 10 DPI. One point to be noted here is that the days on which CTs were taken were not consistent among patients. Also, data is not available for every day post-infection (DPI). For example: one patient has 2,4, 8 DPI CT scans while another one has 5, 24, 47 DPI. The results from [26], provide us with the total percentage of opacity (due to GGOs and consolidation) in the lung. They are referred to as “lung opacities”. We use the following method to calculate the volumes of each individual lesion in each patient. All the patient data and analyses are available in Data Repository.

#### 4.1.1 Lung Segmentation and Volume calculation

The CT scans are available in the NIFTI format. We use the tool Slicer (Version 4.11) [95, 96] that has a lung analyzer module that segments the lung area and calculates the lung volumes from CT scans. A step-by-step tutorial can be found in [97].

#### 4.1.2 Lesion Identification and volume calculation

Our radiologist co-authors interpreted the CT scans and guided our manual annotation of the lesion from the scans. The labeled scans were later segmented using computer vision-based approaches [98]. From the total lesion segmentation, we extracted the individual lesions using the Python Library Connected Components 3D [99]. At the end of this step, we have individual lesion analyses from every patient at every timepoint.

#### 4.1.3 Visualization

After the extraction of lung and lesion segments, we overlay the lesions on the lungs using VTK library [100] and visualize them using Paraview [101]. All the calculation and visualization are done in 3D.

#### 4.1.4 Data interpolation and Smoothing for Individual Lesion Analysis

Since CT scans were not acquired every day for every patient, we track individual lesions for the first 10 days. After extracting individual lesion volumes, we organize them by day post-infection (Figure 4a). For each day with available data, the median lesion volume is computed across patients to obtain a representative central tendency, and the standard deviation is calculated to quantify inter-patient variability [62]. To account for missing timepoints (0,1,6) in the longitudinal dataset, linear interpolation was applied across the full range of observed days to generate a continuous daily volume trajectory [102]. The interpolated median volume curve was subsequently smoothed using a onedimensional Gaussian filter with a standard deviation of *σ* = 2 to reduce noise while preserving the underlying temporal trend of lesion growth [103]. The resulting smoothed volume trajectory, together with the interpolated median volumes and the original individual volumes, is illustrated in Figure 4b. The median individual lesion growth rate (Figure 4c) is derived from this median volume. This is the first metric to compare with SIMALI inflammation growth rate.

#### 4.1.5 Lung Lesion Burden Metric

Lung Lesion Burden (LLB) is defined as the ratio of total lesion volume to total lung volume, and serves as an established indicator of disease severity in CT imaging [64, 65]. It provides a normalized, scale-independent metric for consistent comparison across patients, time points, and computational models with different simulation sizes.

For patient CT data, LLB at each timepoint is computed as:

(1) $${\text{LLB}}_{\text{patient}}\left(t\right)=\frac{V_{\text{lesion}}\left(t\right)}{V_{\text{lung}}}\times 100\%$$

where *V*_lesion_(*t*) is the total segmented lesion volume at day *t* and *V*_lung_ is the total lung volume for the same patient, both calculated from CT scans. This definition is consistent with CT assessment methods established in the COVID-19 imaging literature [64].

For SIMALI simulations, LLB is computed from the 3D VTK output files generated at each simulation timestep. Each VTK file stores the cells containing the inflammation concentration which is used as the proxy for lesion/damage. LLB is quantified using the cell with inflammation burden, defined as the fraction of the total simulation domain occupied by inflammed cells at each timepoint.

(2) $${\text{LLB}}_{\text{SIMALI}}\left(t\right)=\frac{N_{\text{inflammation}}\left(t\right)}{N_{\text{total}}}\times 100\%$$

where *N*_total_ is the total number of cells or voxels in the simulation domain and *N*_inflammation_ is the number of cells with inflammation.

##### Scaling for patient comparison:

Since the SIMALI simulation domain (*V*_sim_) is smaller than the total lung volume in CTs (*V*_lung_ ≈ 4000–6000 cm^3^) [26], direct comparison of absolute LLB values requires normalization. Patient LLB is scaled by the ratio of the simulation domain volume to the average patient lung volume:

(3) $${\text{LLB}}_{\text{patient},\text{scaled}}\left(t\right)={\text{LLB}}_{\text{patient}}\left(t\right)\times \frac{V_{\text{sim}}}{{\bar{V}}_{\text{lung}}}$$

where ${\bar{V}}_{\text{lung}}$ is the average patient lung volume across the cohort. Figure A5 shows example of scaling patient burden from CTs to *V*_sim_ = 0.7 cm^3^, corresponding to the 600 × 600 × 600 voxel simulation domain at a voxel resolution of 0.015 mm per side. This scaling preserves the temporal progression of patient LLB while normalizing its magnitude to the simulation domain size. This facilitates direct quantitative comparison between SIMALI predictions and patient CT measurements.

### 4.2 SIMALI Description and Parameter Specification

For SIMALI, we updated the cell types from the generic SIMCoV epithelial cells with a diameter of 5μm to alveolar cells with a diameter of 15μm. This change effectively reduced the spatial and temporal resolution by a factor of three while increasing the simulation dynamics by three-fold compared to SIMCoV [19]. Timesteps are sampled every 480 simulation steps, corresponding to 1 day post-infection (DPI). Given this single-cell-to-single-cell conversion, we assume that our biological parameters remain valid when applied to the larger cell size, allowing each grid point to represent 15μm. Each alveolus is modeled with a diameter of 300μm [1, 55], and we assume each side of the alveolar sac is 1500μm, resulting in 100 grid points across each side of the sac in our model. Consequently, each sac (3D) contains ≈ 125 alveoli. The source code is available at https://github.com/htasnim/SIMALI.

The structures and cells in the simulation are:

- Air: Each alveolus is filled with air.
- Alveolar Epithelium: This forms the outer layer of the sac. There are 2 types of cells:
  - Type I Pneumocytes: They form 95% of the alveolar surface and are responsible for the gas exchange [1, 50]. These cells are non-infectable.
  - Type II Pneumocytes: 5% of the surface are formed with these infectable cells.
- Inside of the sac has multiple alveolar structures. Each alveoli is like a grape with air inside and has alveolar epithelium outside [1].
- The space between the alveolar sacs is called lung tissue in this work.

In SIMCoV [19], there were four main components: epithelial cells, T cells, virions, and inflammatory signals (Figure A2). In our proposed SIMALI model, the epithelial cells have been expanded into four distinct types: air, infectable and non infectable epithelial, and lung tissue. The diffusion parameters of these cells types are summarized in Table 1. Based on the default COVID-19 parameter values from SIMCoV [19] Table 2, the corresponding changes in key parameters for the SIMALI model are summarized in Table 2.

## Supplementary Material

1

## Figures and Tables

**Table 1:** Alveolar Cell Types with Infectivity and Diffusion Coefficients

Cell Type	Infectivity	Virion Diffusion	Inflammation Diffusion
Air	Non-Infectable	1	1
Type I pneumocyte	Non-Infectable	0.05	0.3
Type II pneumocyte	Infectable	0.05	0.3
Lung Tissue	Non-Infectable	0.0001	0.0006

**Table 2:** Changes in the derived parameters for the SIMALI model. Parameters highlighted in yellow indicate new diffusion parameters introduced in SIMALI that are not present in SIMCoV[19]. Abbreviations: num ts, number of timesteps; per ts, per timestep; t.f., time factor; N/A, not applicable.

Parameter	Occurrence	SIMCoV	SIMALI	Calculation
Incubation Period	num ts	480	160	*x* × (1*/*3)
Apoptosis Period	num ts	180	60	*x* × (1*/*3)
Expressing Period	num ts	900	300	*x* × (1*/*3)
Infectivity	per ts	0.001	0.003	Probability t.f. ×3
Virion Production	per ts	1.1	3.3	Additive t.f. ×3
Virion Clearance	per ts	0.004	0.01195	1 − (1 − *x*)^3^
Virion Diffusion (Epithelial)	per ts	0.15	0.05	(*x* = 15, *t* = 180)[21]
Virion Diffusion (Air)	per ts	N/A	1.0	N/A
Virion Diffusion (Lung Tissue)	per ts	N/A	0.0001	N/A
Inflammatory Signal Production	per ts	1.0	1.0	No change (max = 1.0)
Inflammatory Signal Decay	per ts	0.01	0.0297	1 − (1 − *x*)^3^
Inflammation Diffusion (Epithelial)	per ts	0.15	0.3	(*x* = 15, *t* = 180)[21]
Inflammation Diffusion (Air)	per ts	N/A	1.0	N/A
Inflammation Diffusion (Lung Tissue)	per ts	N/A	0.0006	N/A
Antibody Period	num ts	5760	1920	*x* × (1*/*3)
T cell Generation Rate	per ts	105000	315000	Additive t.f. ×3
T cell Initial Delay	num ts	10080	3360	*x* × (1*/*3)
T cell Vascular Period	num ts	5760	1920	*x* × (1*/*3)
T cell Tissue Period	num ts	1440	480	*x* × (1*/*3)
T cell Binding Period	num ts	10	3	*x* × (1*/*3)

## Data Availability

Data Repository.

## Code availability

https://github.com/htasnim/SIMALI.
