# Supplementary material for: Spatial Immune Model of Alveolar Lung Infection (SIMALI) Identifies Structural Determinants of Lung Inflammation
Source: Res Sq. 2026 Jun 24:rs.3.rs-9986593. Preprint. [Version 1] doi: 10.21203/rs.3.rs-9986593/v1 (PMC13321255; doi:10.21203/rs.3.rs-9986593/v1)
Supplement: 1 [file NIHPPrs9986593V1-supplement-1.pdf]

Appendix A Supplementary Figures

Table A1 Simulation results at varying percentages of infectable cells out of 1 million total cells.

	Number of infectable cells (out of 1,000,000 total cells)					
**Run**	**1%**	**10%**	**25%**	**50%**	**75%**	**100%**
R1	132	1,288	3,327	6,712	9,981	13,306
R2	126	1,374	3,362	6,795	10,111	13,483
R3	133	1,312	3,265	6,622	9,972	13,252
R4	116	1,244	3,198	6,543	9,778	12,990
R5	129	1,259	3,222	6,439	9,669	12,912

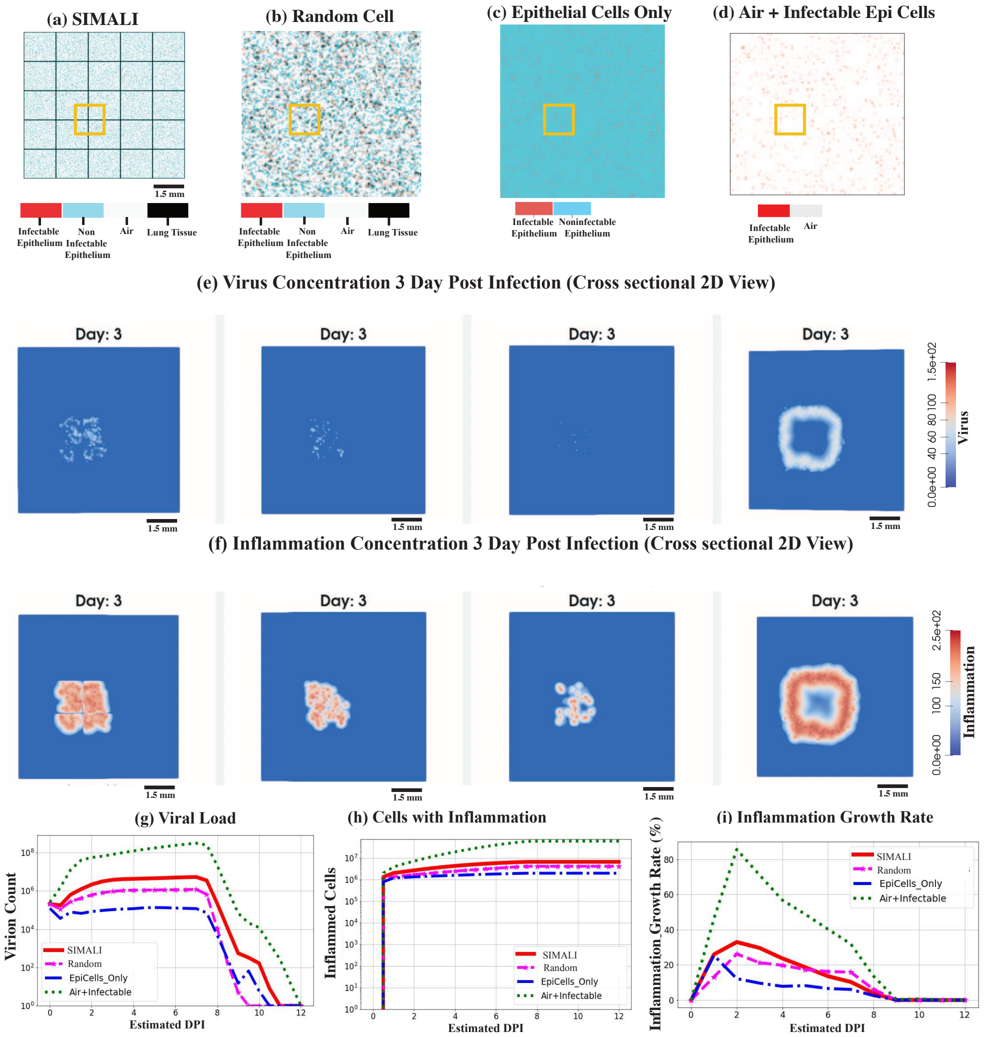

Fig. A1 Effect of alveolar sac structure and cell types in simulations for virus and inflammation diffusion. Starting with (a), SIMALI configuration with sac structure containing air, infectable, non-infectable epithelial cells and lung tissue; we progressively remove structural and cellular components for comparison. (b) represents a random distribution of the four cell types to test the effect of only varying the spatial arrangement of the same cells as (a). (c) removes lung tissue and air to compare only a solid 3D simulation with infectable and noninfectable epithelial cells to compare to the original SIMCoV model. (d) shows elevated diffusion if only infectable cells are distributed in the air, removing the effect of all non-infectable cells and lung tissue. Simulation dimensions are $600 \times 600 \times 600$ voxels, equating to approximately 0.7cm^3 in volume; run for 12 DPI with T cell arrival at 7 DPI. (e) and (f) compare virus concentration and inflammation patterns at 3 DPI across the spatial structures in (a) - (d). The temporal dynamics of viral load, inflammation count, and inflammation growth rate are compared in plots (g–i). Video demonstration is available here.

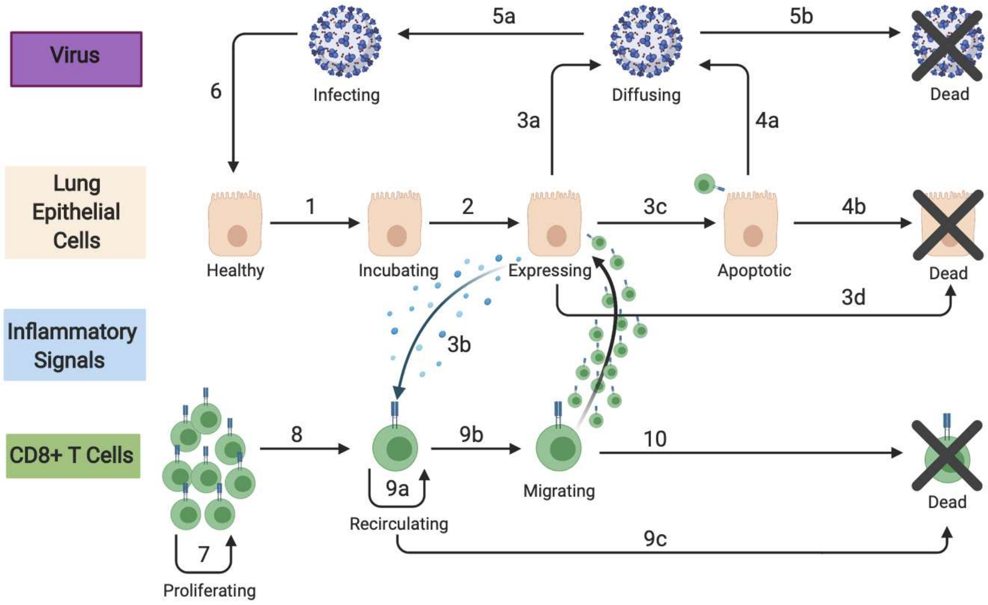

Fig. A2 SIMCoV model components and their interactions. Epithelial and T cells are represented as agents; virions and inflammatory signals are represented as concentrations. Reprinted from [19]

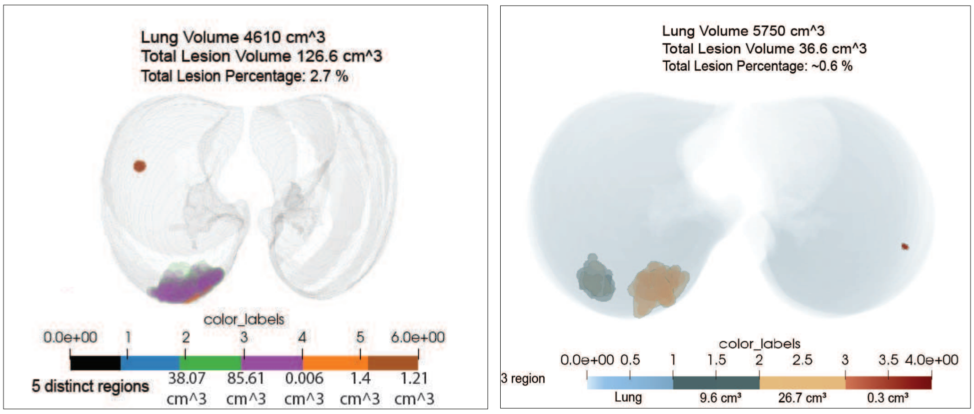

Fig. A3 3D spatial visualization of the infected lung with lesions from sample COVID patients from datasets [92] and [94]

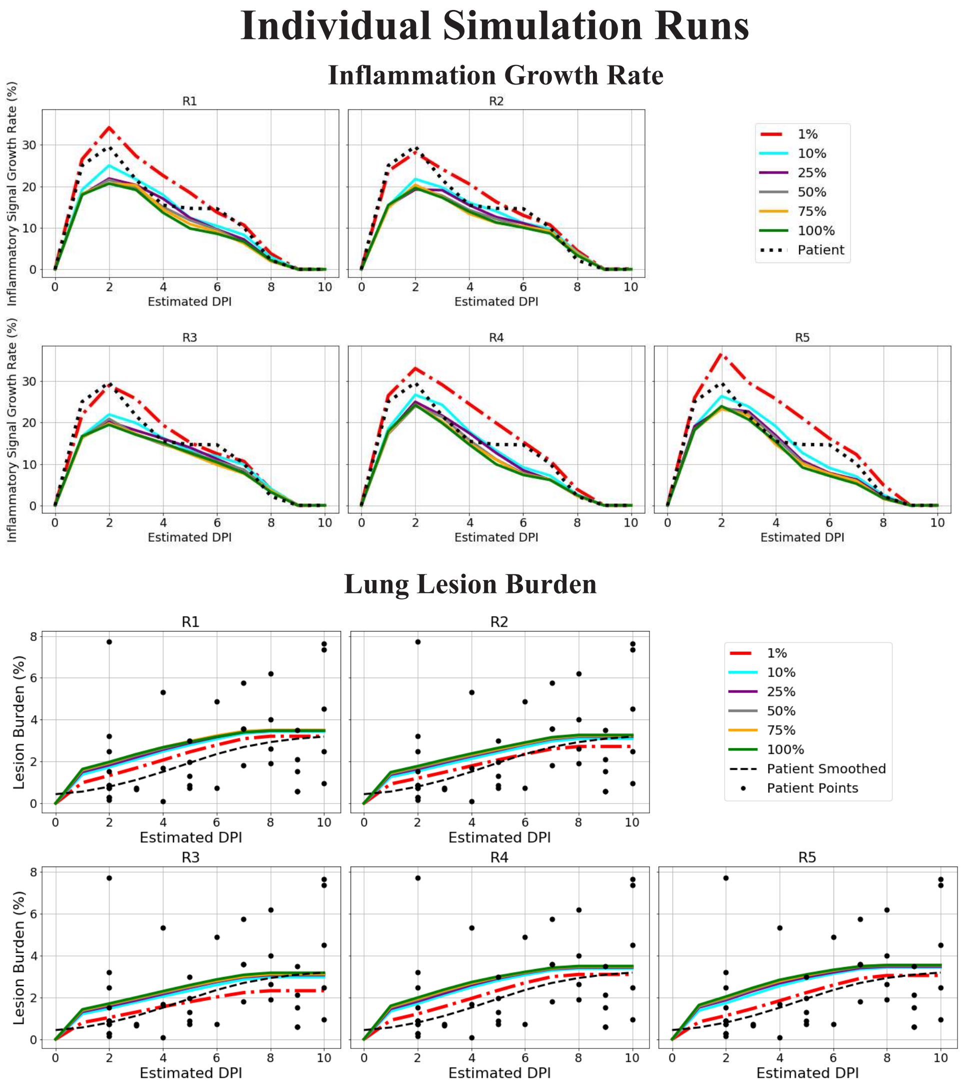

Fig. A4 Individual simulation runs across varying initial infection levels show robustness of SIMALI. Inflammation growth rate (top two rows) and lung lesion burden (bottom two rows) are shown for five independent simulation runs (R1–R5) at five random spatial locations with initial infection levels of 1% (red), 10% (cyan), 25% (purple), 50% (grey), 75% (yellow), and 100% (green) of infectable cells. The number of cells is summarized in Table A1. All simulations use a $600 \times 600 \times 600$ voxel domain $\approx 0.7cm^3$ and run for 10 DPI with T cell arrival at 7 DPI. The Patient median inflammation growth rate (black dotted line) and smoothed LLB (black dashed line) with individual patient data points (black dots) are shown for comparison. The consistency across R1–R5 demonstrates that SIMALI results are robust to stochastic variation in the spatial placement of infection.

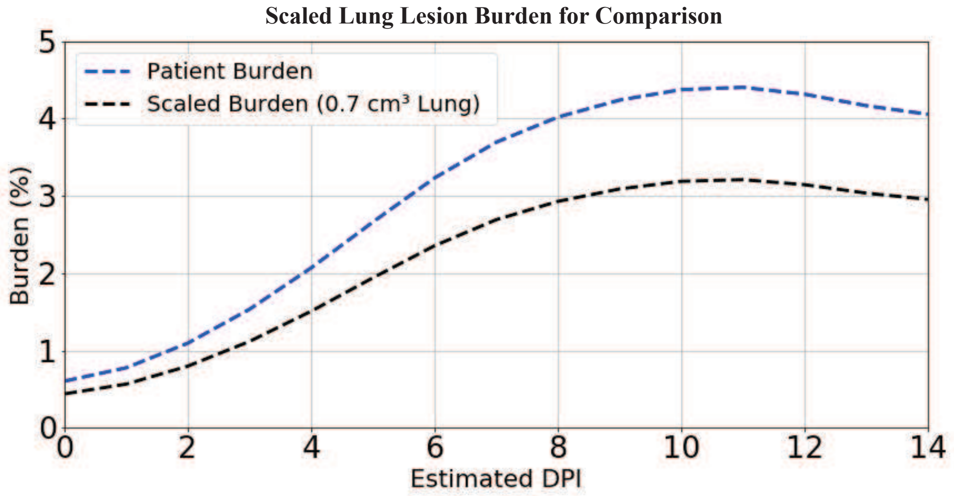

Fig. A5 Scaled lung lesion burden for direct comparison between patient CT data and SIMALI simulations. Patient lung lesion burden (LLB) values (blue dashed line) from the longitudinal CT dataset [26] are scaled by the ratio of the SIMALI simulation domain volume ($0.7cm^3$, black-dashed), enabling direct comparison despite the difference in physical scale between the simulation domain and the full human lung.
